# Therapeutic and Prognostic Potential of G Protein‐Coupled Receptors in Lung Adenocarcinoma: Evidence From Transcriptome Data and In Vitro Experiments

**DOI:** 10.1111/crj.70080

**Published:** 2025-05-13

**Authors:** Feiyan Yang, Jianye Yang, Guobiao Yang, Ya Zhang

**Affiliations:** ^1^ Department of Respiratory and Critical Care Medicine Affiliated Hospital of Shaoxing University (The Shaoxing Municipal Hospital) Shaoxing China

**Keywords:** G protein‐coupled receptors, immune infiltration, lung adenocarcinoma, lung cancer, prognosis

## Abstract

**Background:**

G protein‐coupled receptors (GPCRs), the largest family of cell‐surface molecules involve in various signal transduction, have recently been recognized as important drivers of cancer. However, few studies have reported on the potential of GPCRs as therapeutic targets or biomarkers in lung adenocarcinoma (LUAD).

**Methods:**

The expression profiles and clinical data of LUAD in the GSE30219 and GSE18842 datasets of the Cancer Genome Atlas were analyzed. LUAD‐associated module genes were screened utilizing weighted gene co‐expression network analysis (WGCNA). Prognostic signature genes were identified by univariate Cox survival analysis, LASSO regression, and multivariate Cox regression analyses. The immune status was evaluated and drug sensitivity was determined, conducting in vitro experiments for validation.

**Results:**

Patients with LUAD exhibited lower GPCR score than the controls, and 38 dysregulated GPCRs were identified by screening with differential analysis and WGCNA module genes. An optimal prognostic signature was identified, including OR51E1, LGR4, ADRB1, ADGRD1, and ADGRE3. The model established based on these five genes harbored moderate predictive performance for the survival of patients with LUAD. The risk score was negatively correlated with the infiltrating levels of multiple immune cells, including M2 macrophages, myeloid dendritic cells, and neutrophils, but positively correlated with fewer immune cells, such as Th1/Th2 CD4 + T cell. ADGRE3 and OR51E1 expression was positively correlated with drug sensitivity, including to cisplatin, ribociclib, and pevonedistat. Silencing OR51E1 inhibited the malignant cytological behaviors of LUAD cells.

**Conclusion:**

GPCRs demonstrated prognostic potential in LUAD, with five genes identified as potential therapeutic targets and prognostic biomarkers for LUAD.

## Graphical abstract

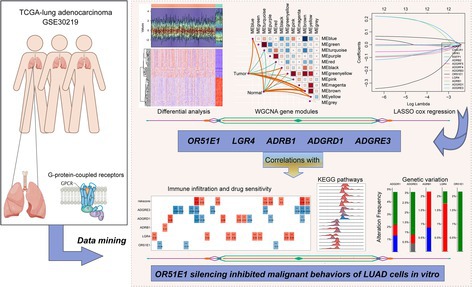

## 1 Introduction

Lung cancer is the most frequently diagnosed malignancy and the leading cause of mortality caused by cancer globally [1]. Based on 2022 global cancer statistics, approximately 12.4% (almost 2.5 million) of all newly diagnosed cancers and 18.7% (1.8 million) of all cancer‐related mortality globally can be attributed to lung cancer [1]. As the most common pathological subtype of lung cancer, lung adenocarcinoma (LUAD) is responsible to approximately 40% of all lung cancers [2]. Treatment options for LUAD rely on factors such as tumor stage, progression, and the overall health of patients. Early‐stage patients are primarily treated surgically, while advanced and metastatic patients require systemic therapy [3, 4], highlighting the requirements of individualized treatment. Recently, although advances in diagnosis and treatment have improved the survival of patients with LUAD to an extent, the prognosis remains distressingly poor due to tumor recurrence and chemotherapy resistance [5, 6, 7]. Hence, to further improve the overall survival of these patients, there is a need to identify novel therapeutic targets and develop specific predictive methods for prognosis.

Currently, studies have been increasingly dedicated to identifying genes or pathomic signatures as biomarkers or therapeutic targets for the diagnosis and prognosis of lung cancer [8, 9]. This includes G protein‐coupled receptors (GPCRs), the largest family of cell‐surface molecules that transduce extracellular signals into a wide range of physiological effects to maintain homeostasis [10]. GPCRs constitute the largest and the most successful family of druggable genes in the human genome, and have been used as targets for 30% of approved drugs currently on the market [11, 12]. GPCRs have been recently recognized as important drivers of cancers [13]. The normal physiological functions of GPCRs are frequently hijacked by malignant cells to impact oncogenesis from multiple aspects by modulating tumor growth, metastasis, angiogenesis, immunological surveillance evasion, and drug resistance [14, 15]. Therefore, revealing the associations between GPCRs and tumor development could provide insights into considered the mechanism of oncogenesis and metastasis, opening up new directions for tumor prevention and treatment [16, 17, 18, 19]. Nevertheless, at present, studies exploring the potential of GPCRs as therapeutic targets or biomarkers in LUAD are scarce.

This study sought to elucidate the precise roles of GPCRs genes in LUAD. Firstly, GPCR genes that were dysregulated in LUAD were screened by intersecting with the genes identified by both differential analysis and weighted gene co‐expression network analysis (WGCNA). Next, machine learning methods were employed to identify the optimal prognostic signature to develop GPCR‐based predictive model, which contributed to stratify patients with LUAD into diverse risk groups with different immune statuses, drug sensitivities, and molecular pathways. Lastly, the expression pattern, genetic variations, and involved oncogenic pathways of key prognostic genes in LUAD were revealed to explore their possible mechanism of actions in LUAD.

## 2 Methods

### 2.1 Data Source

The RNA‐sequencing (RNA‐seq) and clinical phenotype data of LUAD in the Cancer Genome Atlas (TCGA) database were downloaded from the UCSC Xena (accessed on July 2024). The TCGA‐LUAD cohort comprised of 524 tumor and 59 normal samples, which was used as a discovery cohort in this study. Among the 524 LUAD samples, 511 sample harboring complete survival data was used for prognostic analysis. GSE30219 dataset that contained the gene expression profiles and survival data of 85 LUAD and 14 normal samples were downloaded from the Gene Expression Omnibus (GEO) database. This dataset was employed for analysis verification. In addition, the GSE18842 dataset, comprised of 45 normal and 46 tumor samples in GEO database, was downloaded for validating the expression of core genes. A list of GPCR genes was retrieved from a previous study [20].

### 2.2 Gene Set Variation Analysis (GSVA)

To quantify the overall GPCR score, GSVA was conducted for each sample to calculate an enrichment score with the GPCR genes used as the enrichment background by means of a GSVA package (version 1.50.1). Thereafter, the enrichment score was compared across LUAD and controls.

### 2.3 Differential Expression Analysis

Differential analysis across LUAD and controls was performed utilizing the DESeq2 (version 1.42.1) package, followed by Benjamini and Hochberg multiple testing. Genes that differentially expressed in LUAD compared with the controls were screened by |log2FC| > 1 and adjusted *p* < 0.05.

### 2.4 WGCNA

The 50% genes ranked by the median absolute deviation (MAD) were selected for identifying the LUAD‐associated module genes utilizing the WGCNA package (version v1.72–5). Briefly, a scale‐free network was constructed, and an appropriate soft threshold power (the scale‐free topological fit index *R* ^2^ reached 0.9 for the first time) was determined to make the connections between genes in the network follow the scale‐free networks distribution. Then, highly correlated genes modules were identified by means of gene hierarchical clustering and dynamic tree cutting (minmoduleSize = 100). Next, the correlations between each module eigengene and disease phenotype (LUAD and normal) were calculated to identify LUAD‐associated module genes.

### 2.5 Functional Enrichment and Protein–Protein Interaction (PPI) Network

The involved Gene Ontology (GO) terms and KEGG pathways were enriched by enrichment analysis utilizing clusterProfiler package (version 4.4.4). For PPI network construction, the interactions among protein coding genes were predicted based on the data provided in STRING database (https://string‐db.org/), with an interaction score of 0.4.

### 2.6 Construction and Evaluation of Prognostic Risk Model

Univariate Cox survival analysis was conducted to illustrate the prognostic associations of genes, followed by LASSO regression in the glmnet package (version 4.1–8). Genes with non‐zero coefficients in LASSO regression were further included in multivariate Cox regression model and analyzed using the Step function. The prognostic risk model was then developed to calculate the risk score of each patient with LUAD as follows:

$$\text{riskscore}=\sum_{n=1}^{n}{\text{coefi}}^{*}\mathrm{Xi}$$

where *i* and *X* denote the regression coefficient and expression value of genes, respectively. Subsequently, the LUAD samples were assigned into high‐ and low‐risk groups based on the optimal cut‐off value calculated by the surv_cutpoint function, and survival time was compared across two groups. The predictive performance of the model for predicting 1‐, 3‐, and 5‐year survival was assessed by ROC curves plotted by timeROC (version 0.4).

### 2.7 Evaluation of Immune Status

The infiltrating levels of various immune cells in tissue samples were estimated by the six algorithms provided in the immunedeconv package (version 2.1.0) [21], including ABIS, ConsensusTME, EPIC, quanTIseq, TIMER, and xCell algorithms. Correlation analysis was conducted between immune cells and the risk score. Stromal and immune scores in tumor tissues were further inferred by ESTIMATE algorithms, and the sum of these two scores indirectly reflected the tumor purity.

### 2.8 Gene Set Enrichment Analysis (GSEA)

The dysregulated pathways across two risk groups were explored by GSEA. Briefly, after sorting according to the fold changes of gene expression between the two risk groups, GSEA analysis was performed with the KEGG gene set as enrichment references, and the threshold values were adjusted *p* < 0.05 and |normalized enrichment Score (NES)| > 1.

### 2.9 Analysis of Gene Mutations

The mutation frequency and type of the genes in prognostic model was analyzed using cBioPortal (https://www.cbioportal.org/). In addition, the involvement of genes in the common oncogenic pathways were investigated using the GSCALite (https://guolab.wchscu.cn/GSCA/#/) database.

### 2.10 Drug Sensitivity

Based on the data in Genomics of Drug Sensitivity in Cancer (GDSC) database, the sensitivity of tumor samples to common chemotherapeutics were quantified as the IC50 value using the oncoPredict package (version 0.2) to evaluate the response to chemotherapeutics. The correlations of IC50 values with the risk score and gene expression were further explored by correlation analysis, with a correlation threshold of |*r*| > 0.3 and *p* < 0.05.

### 2.11 Cell Culture and Transfection

Human lung epithelial cell line BEAS‐2B and the two LUAD cell lines A549 and H1975 (BOHUI biotechnology Co. Ltd. Guangzhou, China) were cultured in DMEM or RPMI‐1640 medium supplemented with 10% fetal bovine serum and 1% penicillin–streptomycin in an incubator with 5% CO_2_ and 37°C.

Lentiviral vectors carrying sh‐OR51E1 and corresponding negative control (NC) sequences were constructed to inhibit its expression. The sequences of the shRNA against OR51E1 were as follows: 5’‐GCTAGGTAACTTGACAATCAT‐3′ (sense), and 5’‐ATGATTGTCAAGTTACCTAGC‐3′ (antisense). The obtained lentivirus (1 × 10^8^ TU/mL) was added to the medium of A549 cells at 70%–90% fusion degree. Stable transfected A549 cells were screened with the aid of puromycin after 72 h for infections.

### 2.12 qRT‐PCR

Total RNAs were isolated from cells by Trizol reagent, and were reversely transcribed into cDNA. PCR amplification was then conducted utilizing SYBR Green PCR Master Mix with reaction conditions setting as 95°C for 10 min and 40 cycles of 95°C for 12 s and 60°C for 40 s. A list of the primers is provided in Table S1.

### 2.13 Western Blotting

The cell total proteins were isolated and were quantified by means of the BCA assay. After electrophoretically separating, proteins were transferred onto PVDF membranes, blocked, and then co‐incubated with primary antibodies (anti‐OR51E1 (#SAB4500496), anti‐ADRB1 (#SAB2100064), anti‐ADGRE3 (#SAB4503379), anti‐ADGRD1 (#SAB4501242); Merck, Germany) (anti‐LGR4 (#ab321789), anti‐GAPDH (#ab181602); Abcam, UK) overnight at 4°C, followed by incubation with secondary antibodies for 60 min at dark. Thereafter, the blotting bands were visualized utilizing ECL reagent, followed by film exposure.

### 2.14 CCK‐8 Assay

Cells were inoculated into 96‐well plates (2000 cells/well), and 10 μL CCK‐8 was added to each well at 24, 48, and 72 h, respectively, after cells fully attached to the well. Then, the cells were incubated for another 2 h. The resulting relative cell numbers were measured by the optical density at 450 nm on a microplate reader.

### 2.15 Flow Cytometry

Cells were prepared into cell suspension by digesting with 0.25% Trypsin. For cell cycle detection, the cells were fixed utilizing pre‐cooled 70% ethanol and stained by propidium iodide (PI) away from light. The cells were analyzed on flow cytometer at an excitation wavelength of 488 nm. For the detection of cell apoptosis, the cells were double‐stained using Annexin V‐FITC and PI as per the manual of Annexin V‐FITC apoptosis kit (#C1062S; Beyotime, China), followed by cell analysis utilizing a flow cytometer.

### 2.16 Transwell Assays

Cell invasion was evaluated with the aid of Matrigel‐coated Transwells. Briefly, the cells (200 μL, 1 × 10^5^ cells/mL) were inoculated into the upper chamber of Transwell, with the lower chamber filled with 600 μL of 20% PBS‐contained complete medium. Next, the cells were cultured for 24 h, placed in the lower chamber, fixed with absolute methanol, and stained by crystal violet. Eventually, the invaded cells were counted under three randomly selected fields utilizing ImageJ software.

### 2.17 Wound‐Healing Assay

Cells (4 × 10^5^) were seeded into a 6‐well plate for overnight culture. Then, a 200‐μL pipette tip was scraped along the bottom of the plate to wound the cells. The scraped cells were washed off, followed by imaging at 0 h and 24 h. Finally, cell migration was determined by calculating the cells wounding area using ImageJ software.

### 2.18 Statistical Analysis

Experimental data were displayed as the mean ± standard deviation (SD), and was analyzed using ANOVA or two‐way RM ANOVA (for data from CCK‐8 assay) followed by Tukey's multiple comparison test. *p* < 0.05 indicates statistical significance.

## 3 Results

### 3.1 Involvement of GPCR in the Development of LUAD

**FIGURE 1:**
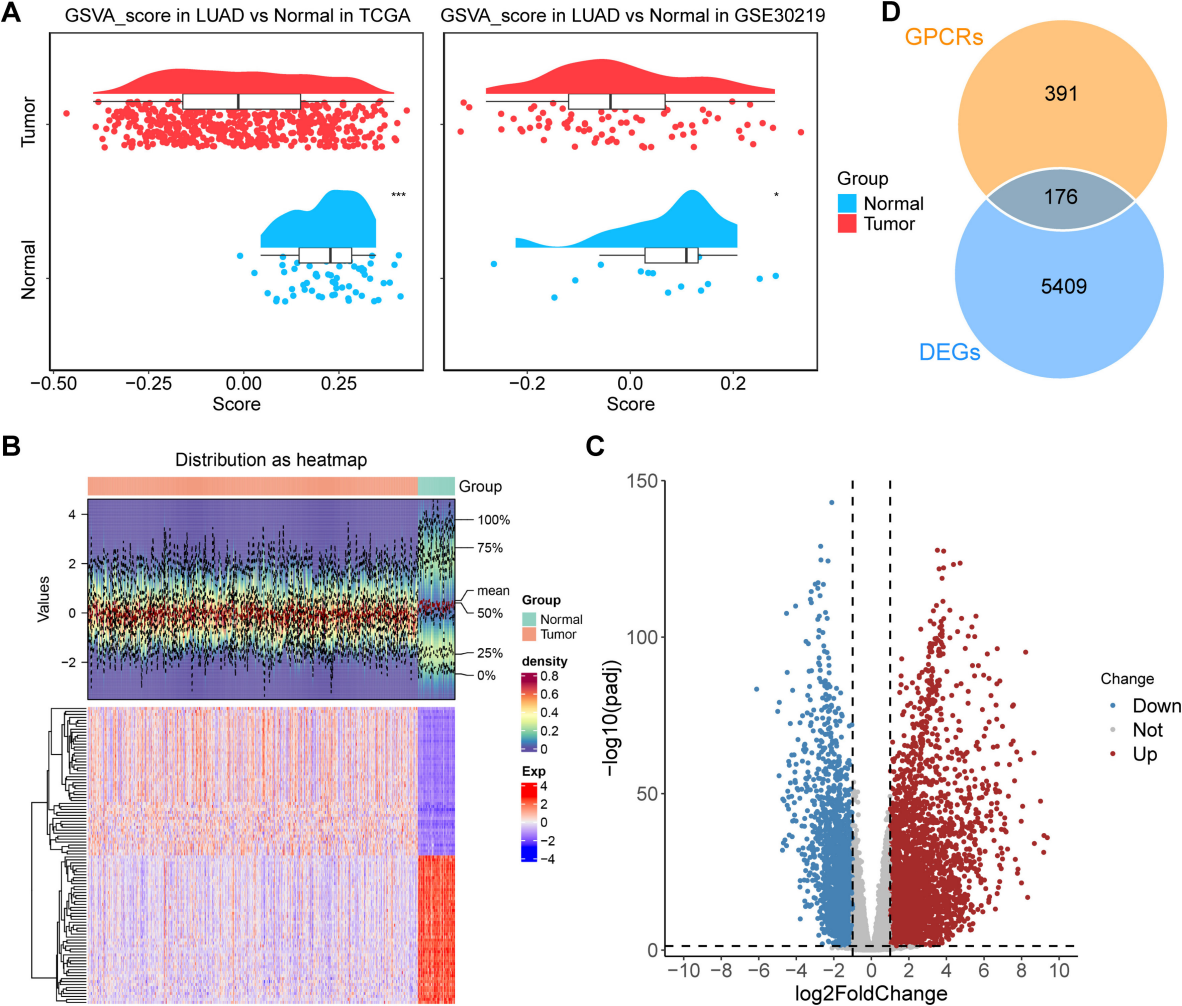
**Dysregulation of GPCRs in LUAD**. (A) GPCR scores of LUAD and normal samples calculated by GSVA. (B–C) Heatmap (B) and volcano plot (C) showing the expression pattern of differentially expressed genes between LUAD and normal samples. (D) Venn diagram showing the overlapped genes between GPCR genes and DEGs.

To illustrate the involvement of GPCR in LUAD, a GPCR score was quantified for each sample by GSVA. Overall, the GPCR score varied greatly between the LUAD and normal samples, with a significantly lower GPCR score in tumor samples in both the TCGA‐LUAD and GSE30219 cohorts (Figure 1A). This implied the dysregulation of GPCR in LUAD. To further explore the expression pattern of GPCR genes in LUAD, differential analysis was conducted between LUAD and normal samples, which revealed 5409 differentially expressed genes (DEGs), including 3458 upregulated and 1951 downregulated genes in LUAD compared with those in the normal samples (Figure 1B,C). After intersecting the 391 GPCR genes with the 5409 DEGs, 176 GPCR genes were found to be differentially expressed in the LUAD samples (Figure 1D).

### 3.2 Screening of LUAD‐Associated Genes by WGCNA

**FIGURE 2:**
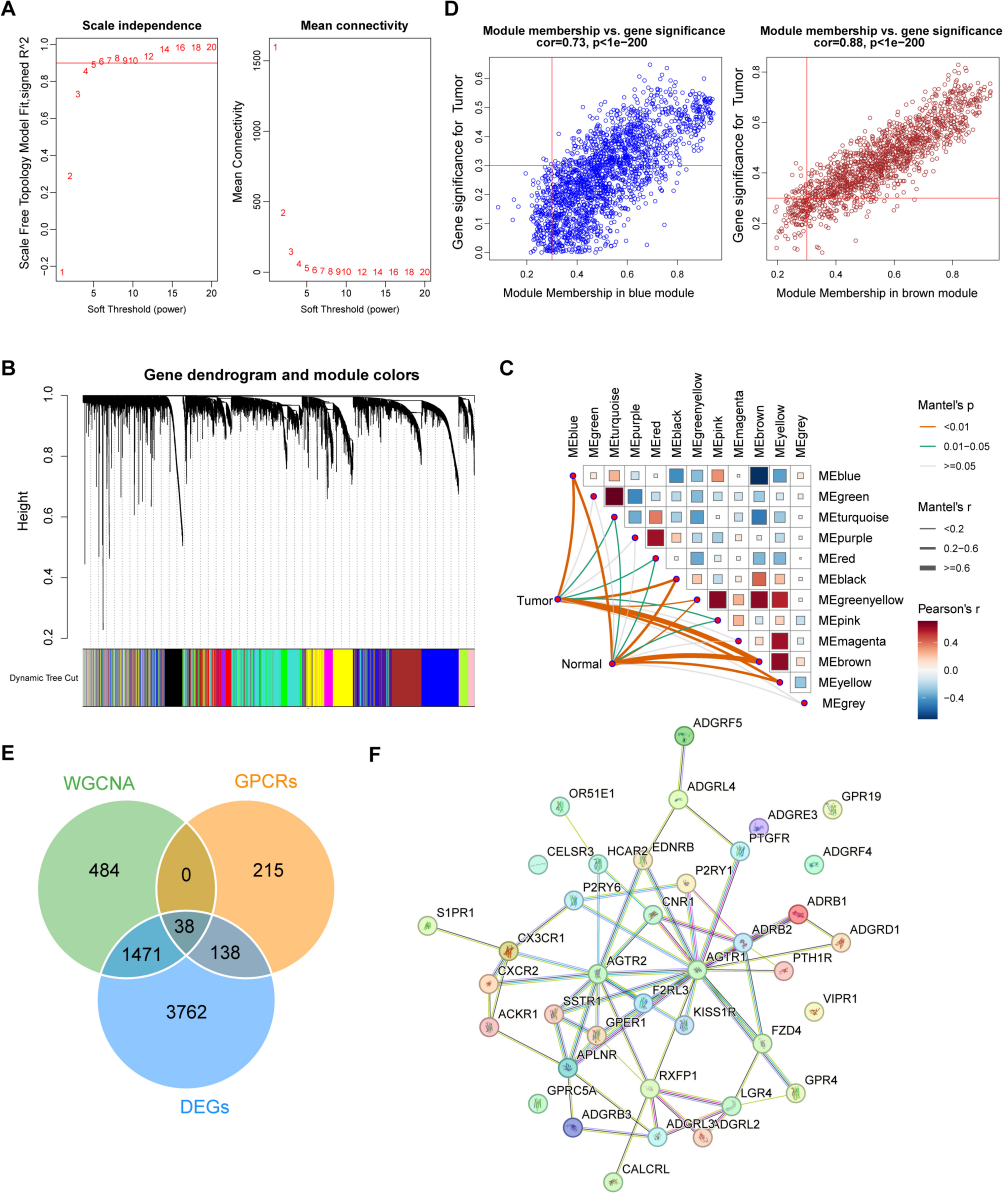
**WGCNA for screening LUAD‐associated module genes**. (A) The scale‐free topology model fit and mean connectivity for determination of soft threshold in WGCNA. (B) Cluster dendrogram showing the gene modules in WGCNA. (C) Module‐trait correlations. (D) Scatterplot of gene significance for weight versus module membership in the blue and brown module. (E) Venn analysis showing the overlapped genes among DEGs, WGCNA module genes and GPCRs. (F) Protein–protein interaction network constructed based on STRING database.

**FIGURE 3:**
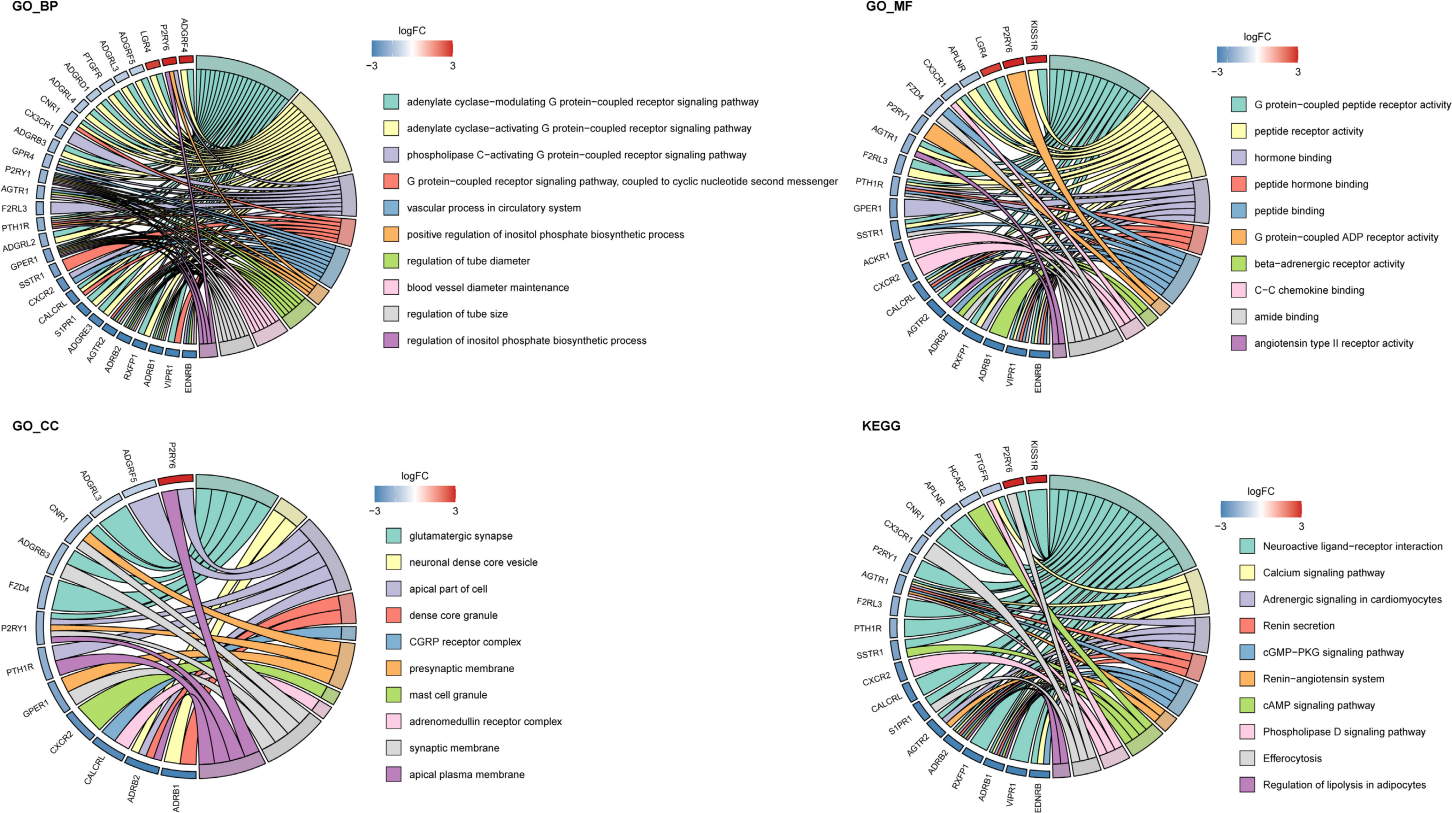
**Functional enrichment results**. The top 10 significantly enriched gene ontology terms (including biological process, cellular component, and molecular function) and KEGG pathways.

WGCNA was conducted to identify highly correlated gene modules. To address the scale‐free networks distribution, a soft threshold power of six was determined, where the scale‐free topological fit index *R* ^2^ reached 0.9 for the first time (Figure 2A). Under this soft threshold, the genes were clustered into 12 modules (Figure 2B), with at least 100 genes in each module. Among the 12 modules, the brown module (*r* = −0.802, *p* < 0.0001) and blue module (*r* = 0.523, *p* < 0.0001) showed the strongest correlation with LUAD in terms of the module‐trait correlations (Figure 2C). Further module membership analysis indicated that 1207 genes in the brown module and 785 genes in the blue module were obtained with the cut‐off values of |gene significance (GS)| > 0.3 and |module membership (MM)| > 0.3 (Figure 2D). Venn analysis revealed 38 overlapped genes among module genes, GPCR genes, and DEGs (Figure 2E). These genes interacted with each other (Figure 2F), suggesting that they may exert their role in a complex. Functional enrichment indicated that these genes were mainly implicated in various GPCR‐related function terms, such as adenylate cyclase‐modulating/adenylate cyclase‐activating/phospholipase C‐activating GPCR signaling pathways, G protein‐coupled peptide receptor activity, G protein‐coupled ADP receptor activity, and CGRP receptor complex (Figure 3). Furthermore, these genes were involved in several KEGG pathways, including neuroactive ligand−receptor interaction and the calcium, cGMP−PKG, and cAMP signaling pathways (Figure 3).

### 3.3 Construction and Evaluation of the CGRP‐Based Predictive Model

**FIGURE 4:**
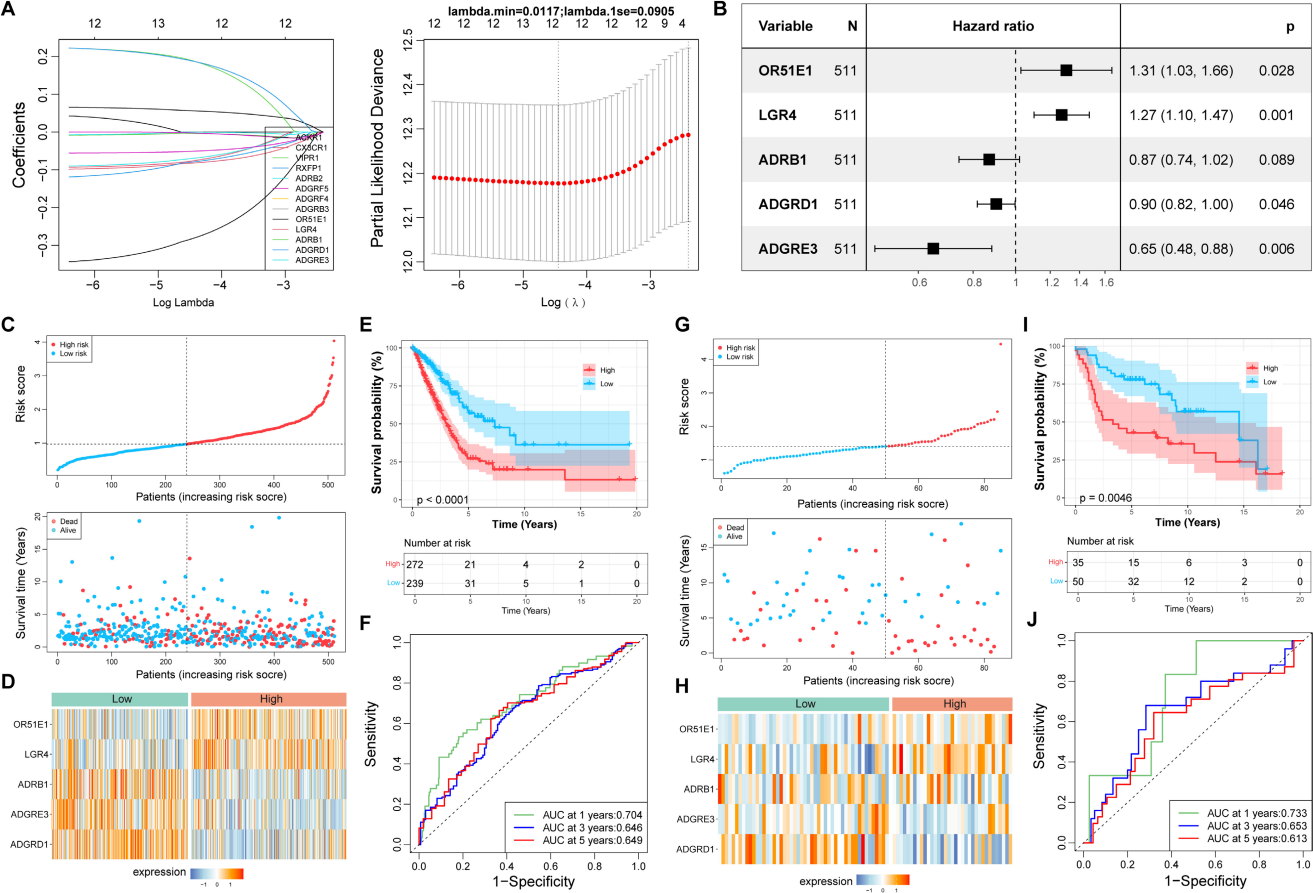
**Construction and evaluation of GPCR‐based prognostic model**. (A) Coefficient distribution and partial likelihood deviance of LASSO regression. (B) Forest plot of the multivariate Cox regression analysis. (C–J) Model construction and evaluation in TCGA (C–F) and GSE30219 (G–J). (C, G) Scatterplot of risk score based on risk groups and survival time. (D, H) Expression pattern of prognostic genes in risk groups. (E, I) Survival curves showing the survival probability between two risk groups. (F, J) ROC curves for evaluating the predictive performance of the model.

The prognostic correlations of these 38 genes were illustrated by univariate Cox survival analysis, and 13 genes were found to be associated with the survival of patients with LUAD. Ten genes were identified as protective factors with a hazard ratio (HR) < 1 and three genes were risk factors with HR > 1. Next, 12 genes (CX3CR1, VIPR1, RXFP1, ADRB2, ADGRF5, ADGRF4, ADGRB3, OR51E1, LGR4, ADRB1, ADGRD1, and ADGRE3) with non‐zero coefficients in LASSO regression were further selected (Figure 4A). These 12 genes were further included in multivariate Cox regression model, and an optimal gene signature was identified by the Step function, including OR51E1, LGR4, ADRB1, ADGRD1, and ADGRE3 (Figure 4B, Table 2). The predictive model established by these five genes categorized patients with LUAD into high‐ and low‐risk groups (Figure 4C). The expression of OR51E1 and LGR4 were gradually elevated, whereas ADRB1, ADGRD1, and ADGRE3 expression were gradually decreased with the risk from low to high (Figure 4D). Survival status differed significantly across these two groups, with a lower survival probability in high‐risk patients with LUAD (Figure 4E). ROC curves demonstrated that there was a moderate predictive power of the model for predicting the 1‐, 3‐, and 5‐year survival for patients with LUAD (Figure 4F). The model was further evaluated by the GSE30219 verification set, and similar findings were observed (Figure 4G–J). The clinical features of patients with LUAD in the two groups were further illustrated. The low‐risk group harbored a high proportion of patients with LUAD with tumors in early‐stage (stage I, 61.9% vs. 47.8%) and with no lymphatic metastasis (N0, 70.7% vs. 60.3%) compared with those in the high‐risk group (Table 1). In terms of therapy, the low‐risk group appeared to harbor a high proportion of patients who were completely responsive to therapy (58.6% vs. 49.6%) and a low rate of progressive disease (10% vs. 14.7%); however, no statistical significance was observed (Table 1). The independent prognostic value of these five genes and clinical factors were further investigated. Tumor stage and the ADGRD1, ADGRE3, and LGR4 genes were identified to be independently associated with the prognosis of patients with LUAD (Figure S1).

**TABLE 1:** Clinical features of patients in two risk groups.

	High	Low	*p*.value
	**(*N* = 272)**	**(*N* = 239)**	
**Gender**			
Female	147 (54.0%)	129 (54.0%)	1
Male	125 (46.0%)	110 (46.0%)	
**Age**			
Mean (SD)	64.7 (10.2)	65.5 (10.0)	0.367
Median [Min, Max]	65.0 [38.0, 88.0]	66.5 [33.0, 87.0]	
Missing	5 (1.8%)	5 (2.1%)	
**Stage**			
I	130 (47.8%)	148 (61.9%)	**0.01**
II	74 (27.2%)	46 (19.2%)	
III	52 (19.1%)	28 (11.7%)	
IV	13 (4.8%)	12 (5.0%)	
Unknown	3 (1.1%)	5 (2.1%)	
**Pathologic_T**			
T1	75 (27.6%)	95 (39.7%)	0.053
T2	159 (58.5%)	115 (48.1%)	
T3	26 (9.6%)	20 (8.4%)	
T4	11 (4.0%)	7 (2.9%)	
Unknown	1 (0.4%)	2 (0.8%)	
**Pathologic_N**			
N0	164 (60.3%)	169 (70.7%)	**0.032**
N1	60 (22.1%)	34 (14.2%)	
Unknown	48 (17.6%)	36 (15.1%)	
**Pathologic_M**			
M0	184 (67.6%)	157 (65.7%)	0.868
M1	13 (4.8%)	11 (4.6%)	
Unknown	75 (27.6%)	71 (29.7%)	
**Death events**			
Mean (SD)	0.474 (0.500)	0.234 (0.424)	**<0.001**
Median [Min, Max]	0 [0, 1.00]	0 [0, 1.00]	
**Therapy**			0.142
Complete remission/response	135 (49.6%)	140 (58.6%)	
Partial remission/response	2 (0.7%)	2 (0.8%)	
Progressive disease	40 (14.7%)	24 (10.0%)	
Stable disease	15 (5.5%)	18 (7.5%)	
Unknown	80 (29.4%)	55 (23.0%)	
**Smoking**			0.952
Mean (SD)	2.82 (1.10)	2.82 (1.04)	
Median [Min, Max]	3.00 [1.00, 5.00]	3.00 [1.00, 5.00]	
Unknown	8 (2.9%)	6 (2.5%)	

**TABLE 2:** The detailed information of the identified core prognostic genes.

Symbol	Gene ID	Biotypes	ENSEMBL	Chr	Start	End	Annotation
ADRB1	153	Protein_coding	ENSG00000043591	Chr10	114 043 866	114 046 904	Beta‐1 adrenergic receptor
OR51E1	143 503	Protein_coding	ENSG00000180785	Chr11	4 643 420	4 655 488	Olfactory receptor 51E1
LGR4	55 366	Protein_coding	ENSG00000205213	Chr11	27 365 961	27 472 790	Leucine‐rich repeat‐containing G‐protein coupled receptor 4
ADGRD1	283 383	Protein_coding	ENSG00000111452	Chr12	130 953 907	131 141 469	Adhesion G‐protein coupled receptor D1
ADGRE3	84 658	Protein_coding	ENSG00000131355	Chr19	14 619 117	14 690 027	Adhesion G protein‐coupled receptor E3

### 3.4 Heterogeneity in Immune Status, Chemosensitivity, and Pathways Between Risk Groups

**FIGURE 5:**
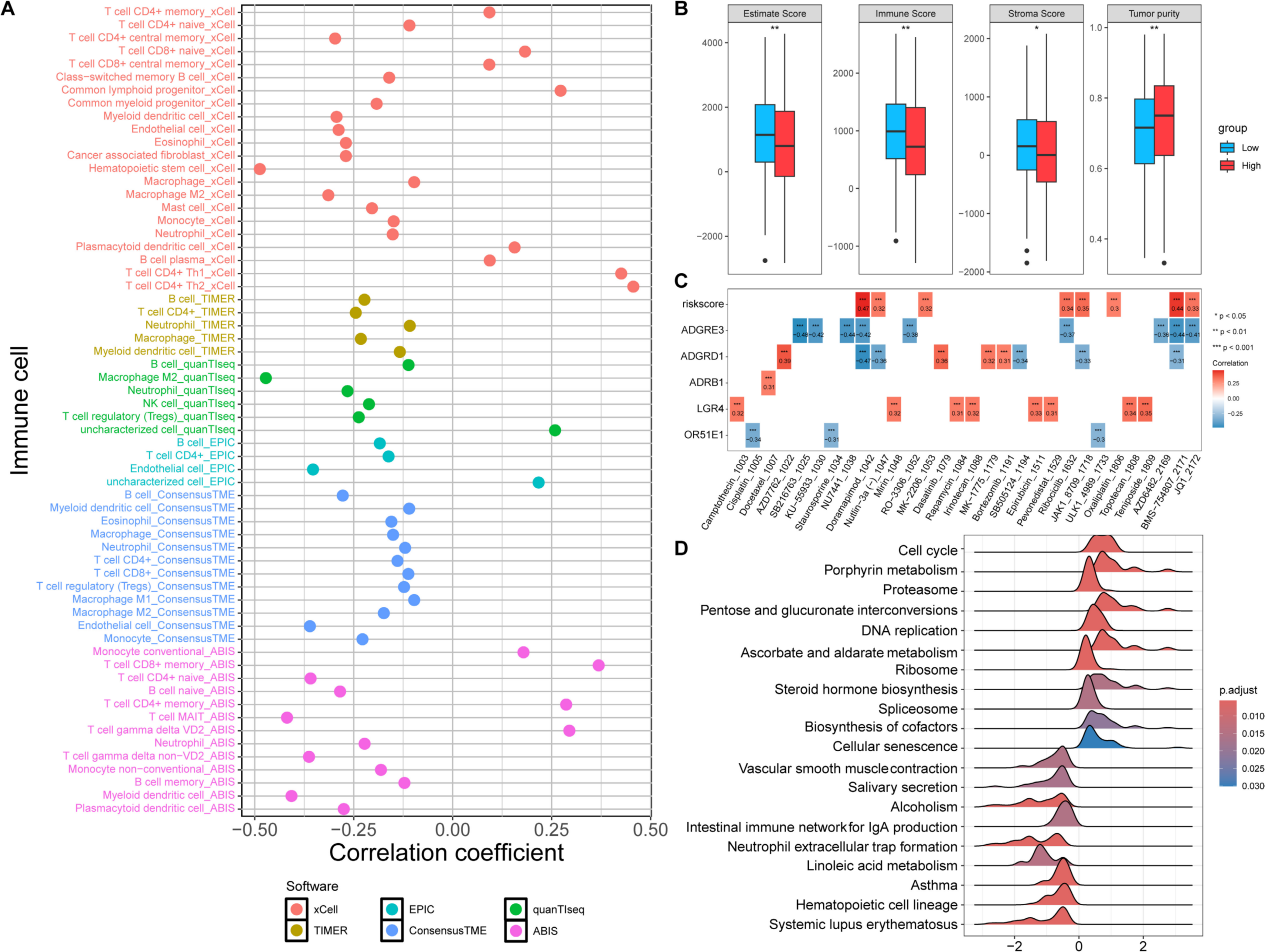
**Differences in immune status, drug sensitivity, and pathways between risk groups**. (A) Results of correlation analysis between infiltrating immune cells and risk score. (B) Boxplots showing the immune, stromal scores, and tumor purity between two risk groups. (C) Correlation between IC50 value to drugs and risk score, as well as gene expression. (D) Significantly altered pathways between risk group analyzed by gene set enrichment analysis.

The infiltrating levels of various immune cells in tumor samples were inferred with six algorithms, followed by correlation analysis with the risk score (Figure 5A). Multiple immune cells exhibited negative correlations with the risk score, including M2 macrophages, myeloid dendritic cells, and neutrophils. By contrast, cells such as Th1 CD4 + T cells, Th2 CD4 + T cells, and memory CD8 + T cells exhibited positive correlations the risk score (Figure 5A). High‐risk patients with LUAD harbored lower stromal and immune cells in tumor tissue but high tumor purity in their tumor tissue (Figure 5B), indicating an immunosuppressive state in high‐risk patients with LUAD. The IC50 value of patients with LUAD to various drugs was estimated to assess their chemosensitivity, and correlations with risk score and gene expression were explored. As displayed in Figure 5C, risk scores were found to be positively correlated with the IC50 to eight drugs. In other words, a negative correlation was observed between the risk score and drug sensitivity. This suggests that high‐risk patients with LUAD may be more likely to benefit from chemotherapy. Furthermore, ADGRE3 and OR51E1 expression was negatively correlated with the IC50 value to drugs (positive correlations with drug sensitivity), such as OR51E1 with cisplatin (*r* = −0.34, *p* < 0.001) and ADGRE3 with ribociclib (*r* = −0.37, *p* < 0.001) and pevonedistat (*r* = −0.37, *p* < 0.001). These results suggest that the expression of these genes may reflect the response to chemotherapy to some extent. The significantly different pathways across the two risk groups were investigated, and the top 20 pathways are displayed in Figure 5D. Several pathways, such as cell cycle, porphyrin metabolism, DNA replication, and steroid hormone biosynthesis, were found to be activated in the high‐risk group, whereas linoleic acid metabolism and neutrophil extracellular trap formation were activated in the low‐risk group.

### 3.5 Expression and Mutations of Prognostic Genes in Patients With LUAD

**FIGURE 6:**
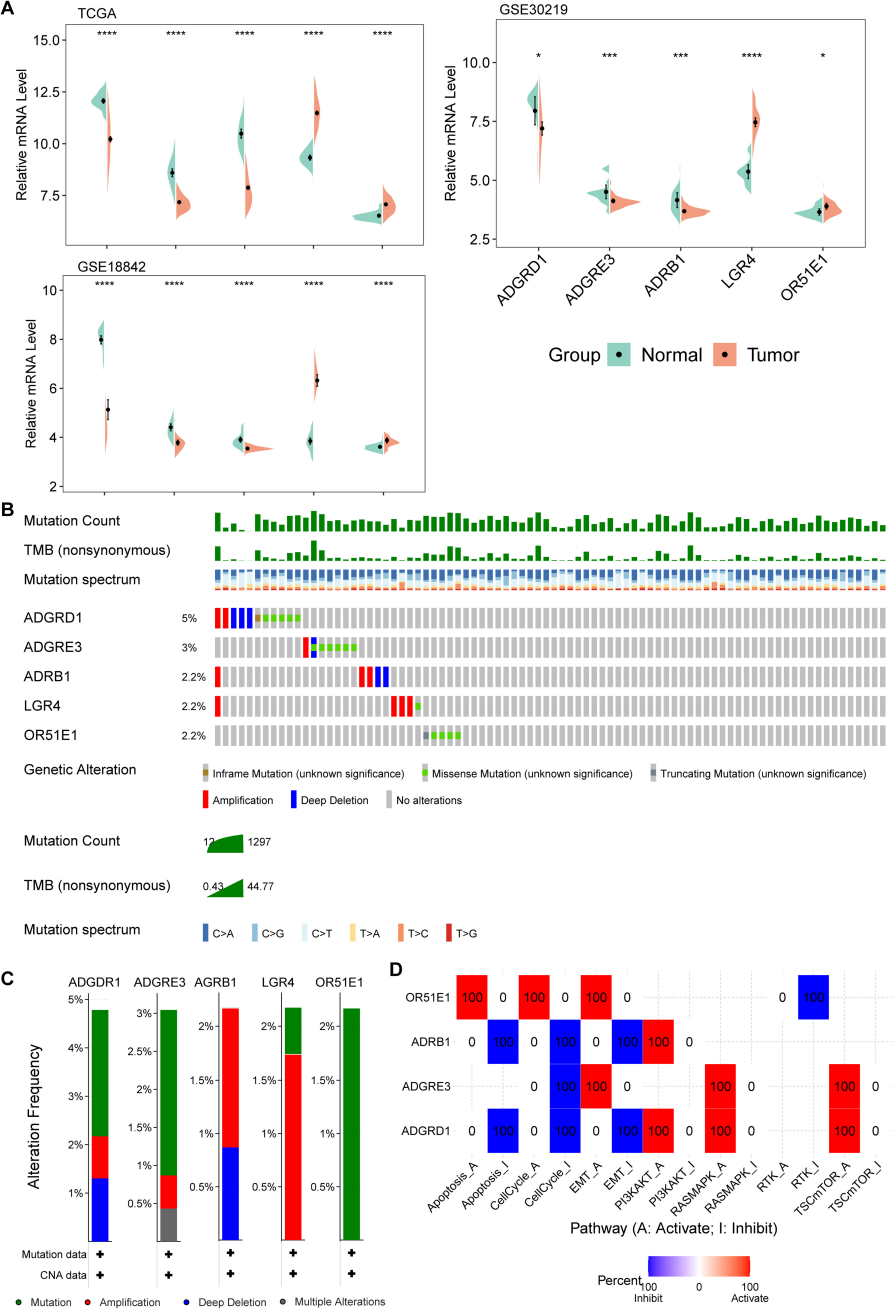
**Expression and genetic variations of prognostic genes**. (A) Expression of five prognostic genes between LUAD and normal samples in TCGA and GSE30219 dataset. (B–C) Frequency and types of genetic variations in prognostic genes in LUAD samples. (D) Correlations between prognostic genes and oncogenic pathways.

In both TCGA‐LUAD, in the GSE30219 and GSE18842 cohorts, the expression of ADRB1, ADGRD1, and ADGRE3 were reduced, whereas the expression of OR51E1 and LGR4 were elevated in tumor samples (Figure 6A). Genetic alteration frequency and the type of these genes were further investigated in the TCGA‐LUAD cohort (Figure 6B,C). The alteration frequency of ADGRD1 was 5%, followed by ADGRE3 (3%). The alteration type included amplification, deep deletion, and missense mutation. ADRB1 and ADGRD1 were associated with the inhibition of apoptosis, cell cycle, and EMT pathways. Inversely, OR51E1 was associated with the activation of apoptosis, cell cycle, and EMT pathways (Figure 6D).

### 3.6 OR51E1 Silencing Inhibited the Malignant Phenotype of LUAD Cells

**FIGURE 7:**
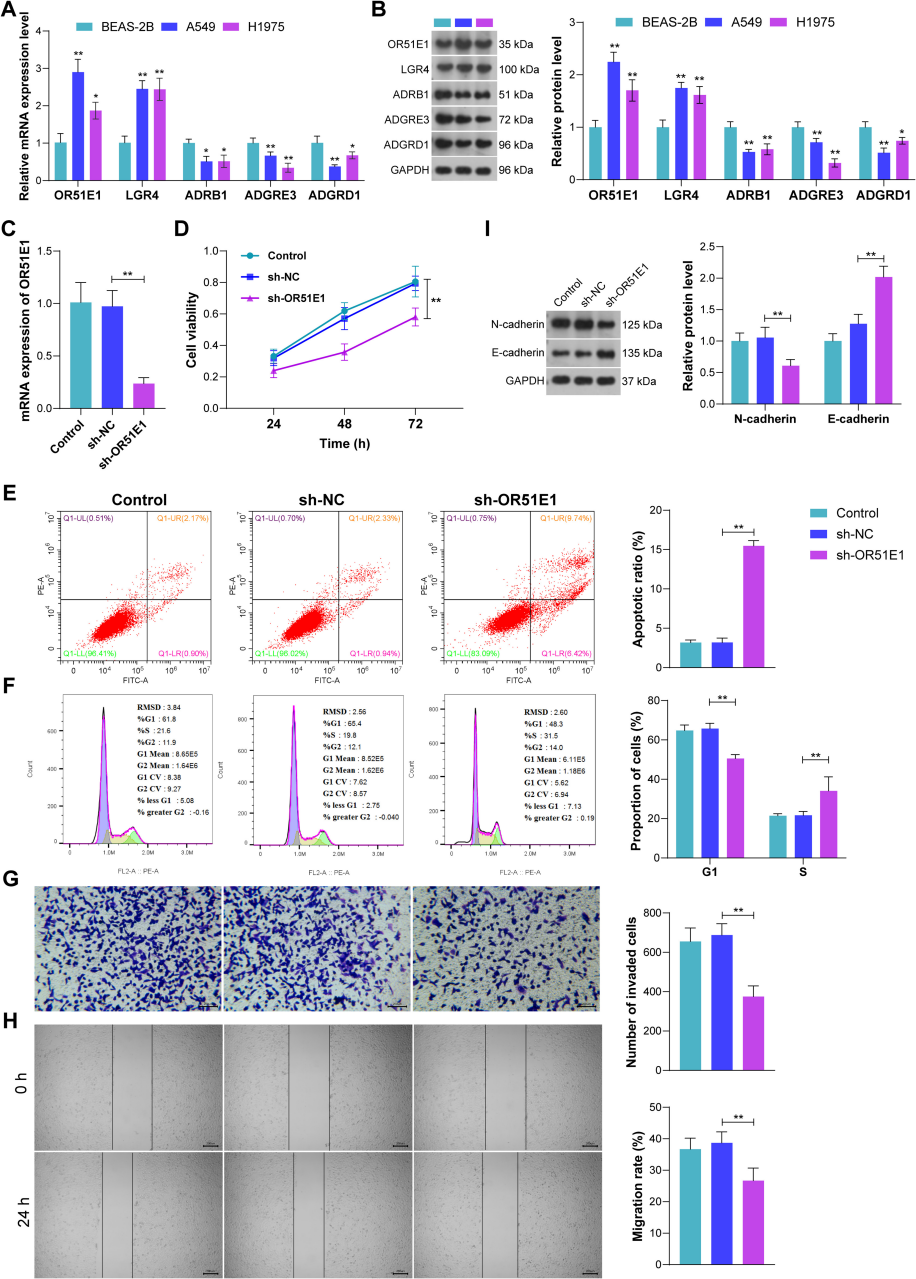
**In vitro experiments**. (A–B) mRNA expression (A) and protein expression (B) of five prognostic genes in normal lung epithelial cell line BEAS‐2B and LUAD cells A549 and H1975. (C) mRNA expression of OR51E1 after transfecting sh‐OR51E1. (D) Cell viability determined by CCK‐8 assay. (E–F) Cell apoptosis (E) and cell cycle (F) determined by flow cytometry. (G) Cell invasion determined by Transwell assay. (H) Cell migration determined by wound‐healing assay. (I) Expression of N‐cadherin and E‐cadherin determined by western blotting. *p < 0.05; **p < 0.01.

Lastly, a series of in vitro experiments were performed to verify the key genes screened in the above analyses. First, the expression of the five genes in two LUAD cells was determined. Consistent with the findings from bioinformatics analysis, mRNA and protein expression of OR51E1 and LGR4 were enhanced in both A549 and H1975 cells compared with a normal lung epithelial cell line BEAS‐2B, while the mRNA and protein expression of ADRB1, ADGRD1, and ADGRE3 were decreased (Figure 7A,B). Among these five genes, OR51E1 was identified as a significant risk factor for LUAD with the highest HR; however, its role in LUAD remains unclear. Hence, we successfully silenced OR51E1 expression in A549 cells (Figure 7C) to explore its actions in LUAD cells. The silencing of OR51E1 markedly inhibited cell viability (Figure 7D) of A549 cells, promoted A549 cells apoptosis (Figure 7E) and S phase arrest (Figure 7F), and inhibited A549 cells invasion and migration (Figure 7G,H). Additionally, the silencing of OR51E1 markedly decreased the expression of EMT markers N‐cadherin and N‐cadherin (Figure 7I). Taken together, these findings suggest that OR51E1 silencing could inhibit the malignant phenotype of LUAD cells.

## 4 Discussion

Despite the fact that GPCRs are the largest signal‐conveying receptor family and regulate a wide variety of physiological processes, their roles in the biology of tumors in humans, particularly in patients with LUAD, are underappreciated. In this context, a bioinformatics analysis was conducted to clarify the potential of GPCRs in LUAD. Based on the 391 GPCR genes retrieved from a previous study [20] and the GSVA method, the GPCR score for each individual in both the TCGA and GSE30219 cohorts was quantified, and significantly lower GPCR scores were observed in the tumor samples. Furthermore, among the 391 GPCR genes, 176 (45%) genes were differentially expressed in the LUAD samples. These results are indicative of the dysregulation of GPCRs in LUAD. Reportedly, the signaling landscape and functional state of a cell could be remarkably altered under the dysregulation of GPCRs and their coupled heterotrimeric G proteins [22]. Hence, it was reasonably inferred that the dysregulation of GPCRs was responsible for the carcinogenesis and progression of LUAD to some extent.

WGCNA is a powerful tool for discovering highly correlated gene co‐expression modules and illustrating the associations of the module genes and clinical traits comprehensively [23], and has been widely used for identifying hub genes or biomarkers in various human diseases [24, 25, 26]. In the current study, LUAD‐associated module genes were screened by means of WGCNA, and the module genes were found to share 38 genes with the dysregulated GPCRs in LUAD. These 38 genes were considered as key GPCRs in LUAD. We further investigated the prognostic value of these 38 genes by machine learning methods, and an optimal prognostic gene signature was identified, including OR51E1, LGR4, ADRB1, ADGRD1, and ADGRE3.

The pathophysiological effects of OR51E1, an olfactory receptor, in the occurrence and progression of tumors have not yet been illustrated. A previous study found that high levels of OR51E1 expression may be a biomarker for diagnosis and therapy in somatostatin receptor‐negative lung carcinoids [27]. LGR4, also denoted by GPR48, belongs to the type B subfamily of rhodopsin GPCRs, which is well recognized for its role in modulating the ability of cells to respond to Wnt signaling [28]. Alterations on Wnt signaling are prominent in human tumors [29]. In NSCLC, tumorigenesis, resistance to therapy, and tumor progression were substantially attributed to Wnt signaling [30]. Based on bioinformatics analysis, Dodin et al. [31] proposed that LGR4 could be a prognostic biomarker in patients with LUAD with KRAS mutation. Dysregulatin on RSPO3 (a R‐spondins ligand of LGR4)‐LGR4 signaling in LUAD with Keap1 deficiency could facilitate tumor aggressiveness [32]. ADRB1 encodes a β1 adrenergic receptor. Evidence is increasingly demonstrating the close associations between β‐adrenergic receptor signaling and immunology of tumors [33, 34]. For example, exhausted CD8 + T cells have been reported to cluster around sympathetic nerves in an ADRB1‐dependent way, and the transformation of T cells towards to an exhausted phenotype could be limited by ablating the β1‐adrenergic signaling during chronic infection [35]. Data from a pan‐cancer set revealed that elevated ADRB1 expression was linked to a worse survival outcome of solid tumors [36]. ADGRD1 and ADGRE3 are adhesion GPCRs, and the dysregulation of various adhesion‐GPCR molecules has been reported to involve the tumorigenesis of different human tumors [37, 38]. Lv et al. observed a reduced expression of ADGRD1 in non‐small cell lung cancer; in particular, ADGRD1 exhibited a strong correlation with the microsatellite instability, mutational burden, and infiltrating immune status in LUAD [39]. Although the specific role of these five genes, particularly OR51E1, ADRB1, and ADGRE3, in LUAD has not been reported, this analysis suggests that ADRB1 and ADGRD1 are associated with the inhibition of apoptosis, cell cycle, and EMT pathways. Inversely, OR51E1 was found to be associated with the activation of apoptosis, cell cycle, and EMT pathways. These findings pave the way for elucidating the precise role played by these genes in LUAD.

The prognostic model developed based on these five genes showed moderate predictive power for the prognosis of patients with LUAD, which contributed to stratify patients with LUAD into high‐ and low‐risk groups. Risk stratification has been demonstrated to show enormous potential in the development of personalized treatments for lung cancer patients [40, 41]. The risk score calculated based on these five genes were closely linked to the infiltrating immune status. A high risk score tended to be associated with an immunosuppressive state in the tumor microenvironment. Furthemore, ADGRE3 and OR51E1 expression was positively correlated with sensitivity to the drugs cisplatin, ribociclib, and pevonedistat, chemotherapeutics commonly used or being investigated in clinical trials for lung cancer [42, 43, 44]. Platinum‐based chemotherapy, in particular cisplatin, is the primary therapeutic strategy in lung cancer [45], and acquired chemoresistance in the period of therapy has emerged as the principal issue for clinicians and the leading cause of failure during treatment [44]. These results suggest that the expression of these genes may reflect the response to chemotherapy to some extent. Taken together, the results highlight the potential of these five genes as prognostic biomarkers for patients with LUAD. However, the results were obtained based on the mining and analysis of public dataset, and further validations using clinical data and experimental investigations are required.

In conclusion, this study demonstrated the dysregulation of GPCRs in LUAD and highlighted the potential of OR51E1, LGR4, ADRB1, ADGRD1, and ADGRE3 as biomarkers and targets in the tumorigenesis and progression of LUAD. A GPCR‐based predictive model was established for the risk stratification of patients with LUAD, thereby contributing to the development of individualized treatment approaches. The in‐depth analysis of the association between GPCRs and LUAD development provided in this study provides insights into the mechanism of oncogenesis and progression, and thus paves the ways for new directions in the prevention and treatment of LUAD.

## Author Contributions

**Feiyan Yang and Ya Zhang:** Conception and design of the research. **Feiyan Yang, Jianye Yang and Guobiao Yang:** Acquisition of data. **Feiyan Yang, Jianye Yang and Guobiao Yang:** Experimental studies. **Feiyan Yang and Ya Zhang:** Analysis and interpretation of data. **Feiyan Yang and Ya Zhang:** Statistical analysis. **Feiyan Yang:** Drafting the manuscript. **Jianye Yang, Guobiao Yang and Ya Zhang:** Revision of manuscript for important intellectual content.

## Ethics Statement

The authors have nothing to report.

## Conflicts of Interest

The authors declare no conflicts of interest.

## Supporting information


**Figure S1.** Identification of independent prognostic factors by multivariate Cox regression.

**Table S1.** Sequences of the primers used in qRT‐PCR. This integrated analysis demonstrates the involvements of G protein‐coupled receptors (GPCRs) in lung adenocarcinoma developments, and proves the potential of five key GPCRs as prognostic biomarkers and targets in lung adenocarcinoma.

**Data S1.** Supplementary Information.

## Data Availability

All data generated or analyzed during this study are included in this article.

## References

[1] F. Bray , M. Laversanne , H. Sung , et al., “Global Cancer Statistics 2022: GLOBOCAN Estimates of Incidence and Mortality Worldwide for 36 Cancers in 185 Countries,” CA: A Cancer Journal for Clinicians 74, no. 3 (2024): 229–263. 38572751 10.3322/caac.21834

[2] C. Qi , J. Ma , J. Sun , X. Wu , and J. Ding , “The Role of Molecular Subtypes and Immune Infiltration Characteristics Based on Disulfidptosis‐Associated Genes in Lung Adenocarcinoma,” Aging 15, no. 11 (2023): 5075–5095. 37315289 10.18632/aging.204782 PMC10292876

[3] F. R. Hirsch , G. V. Scagliotti , J. L. Mulshine , et al., “Lung Cancer: Current Therapies and New Targeted Treatments,” Lancet (London, England). 389, no. 10066 (2017): 299–311. 27574741 10.1016/S0140-6736(16)30958-8

[4] M. G. Kris , L. E. Gaspar , J. E. Chaft , et al., “Adjuvant Systemic Therapy and Adjuvant Radiation Therapy for Stage I to IIIA Completely Resected Non‐Small‐Cell Lung Cancers: American Society of Clinical Oncology/Cancer Care Ontario Clinical Practice Guideline Update,” Journal of Clinical Oncology: Official Journal of the American Society of Clinical Oncology 35, no. 25 (2017): 2960–2974. 28437162 10.1200/JCO.2017.72.4401

[5] Y. Li , B. Yan , and S. He , “Advances and Challenges in the Treatment of Lung Cancer,” Biomedicine & Pharmacotherapy = Biomedecine & Pharmacotherapie. 169 (2023): 115891. 37979378 10.1016/j.biopha.2023.115891

[6] C. Allemani , T. Matsuda , V. Di Carlo , et al., “Global Surveillance of Trends in Cancer Survival 2000‐14 (CONCORD‐3): Analysis of Individual Records for 37 513 025 Patients Diagnosed With One of 18 Cancers From 322 Population‐Based Registries in 71 Countries,” Lancet (London, England). 391, no. 10125 (2018): 1023–1075. 29395269 10.1016/S0140-6736(17)33326-3 PMC5879496

[7] A. A. Thai , B. J. Solomon , L. V. Sequist , J. F. Gainor , and R. S. Heist , “Lung Cancer,” Lancet (London, England). 398, no. 10299 (2021): 535–554. 34273294 10.1016/S0140-6736(21)00312-3

[8] J. Sun , Z. Zhang , S. Bao , et al., “Identification of Tumor Immune Infiltration‐Associated lncRNAs for Improving Prognosis and Immunotherapy Response of Patients With Non‐small Cell Lung Cancer,” Journal for Immunotherapy of Cancer 8, no. 1 (2020): e000110. 32041817 10.1136/jitc-2019-000110 PMC7057423

[9] Y. Zhang , Z. Yang , R. Chen , et al., “Histopathology Images‐Based Deep Learning Prediction of Prognosis and Therapeutic Response in Small Cell Lung Cancer,” npj Digital Medicine 7, no. 1 (2024): 15. 38238410 10.1038/s41746-024-01003-0 PMC10796367

[10] D. Yang , Q. Zhou , V. Labroska , et al., “G Protein‐Coupled Receptors: Structure‐ and Function‐Based Drug Discovery,” Signal Transduction and Targeted Therapy 6, no. 1 (2021): 7. 33414387 10.1038/s41392-020-00435-w PMC7790836

[11] G. P. Schmitz and B. L. Roth , “G Protein‐Coupled Receptors as Targets for Transformative Neuropsychiatric Therapeutics,” American Journal of Physiology. Cell Physiology 325, no. 1 (2023): C17–c28. 37067459 10.1152/ajpcell.00397.2022 PMC10281788

[12] R. Ribeiro‐Oliveira , M. Vojtek , S. Gonçalves‐Monteiro , et al., “Nuclear G‐Protein‐Coupled Receptors as Putative Novel Pharmacological Targets,” Drug Discovery Today 24, no. 11 (2019): 2192–2201. 31520747 10.1016/j.drudis.2019.09.003

[13] P. K. Chaudhary and S. Kim , “An Insight Into GPCR and G‐Proteins as Cancer Drivers,” Cells 10, no. 12 (2021): 3288. 34943797 10.3390/cells10123288 PMC8699078

[14] R. T. Dorsam and J. S. Gutkind , “G‐Protein‐Coupled Receptors and Cancer,” Nature Reviews. Cancer 7, no. 2 (2007): 79–94. 17251915 10.1038/nrc2069

[15] N. A. Zaidman and J. L. Pluznick , “Understudied G Protein‐Coupled Receptors in the Kidney,” Nephron 146, no. 3 (2022): 278–281. 34261071 10.1159/000517355 PMC8758793

[16] J. R. van Senten , T. S. Fan , M. Siderius , and M. J. Smit , “Viral G Protein‐Coupled Receptors as Modulators of Cancer Hallmarks,” Pharmacological Research 156 (2020): 104804. 32278040 10.1016/j.phrs.2020.104804

[17] B. Song , K. Wang , Y. Peng , et al., “Combined Signature of G Protein‐Coupled Receptors and Tumor Microenvironment Provides a Prognostic and Therapeutic Biomarker for Skin Cutaneous Melanoma,” Journal of Cancer Research and Clinical Oncology 149, no. 20 (2023): 18135–18160. 38006451 10.1007/s00432-023-05486-4 PMC11796589

[18] K. Shen , Q. Wang , L. Wang , et al., “Prediction of Survival and Immunotherapy Response by the Combined Classifier of G Protein‐Coupled Receptors and Tumor Microenvironment in Melanoma,” European Journal of Medical Research 28, no. 1 (2023): 352. 37716991 10.1186/s40001-023-01346-6 PMC10504724

[19] R. Khetan , P. Eldi , N. A. Lokman , et al., “Unveiling G‐Protein Coupled Receptors as Potential Targets for Ovarian Cancer Nanomedicines: From RNA Sequencing Data Analysis to In Vitro Validation,” Journal of Ovarian Research 17, no. 1 (2024): 156. 39068454 10.1186/s13048-024-01479-0 PMC11282829

[20] V. Suteau , M. Munier , R. Ben Boubaker , et al., “Identification of Dysregulated Expression of G Protein Coupled Receptors in Endocrine Tumors by Bioinformatics Analysis: Potential Drug Targets?,” Cells 11, no. 4 (2022): 703. 35203352 10.3390/cells11040703 PMC8870215

[21] G. Sturm , F. Finotello , and M. List , “Immunedeconv: An R Package for Unified Access to Computational Methods for Estimating Immune Cell Fractions From Bulk RNA‐Sequencing Data,” Methods in Molecular Biology (Clifton, NJ). 2120 (2020): 223–232. 10.1007/978-1-0716-0327-7_16 32124323

[22] N. Arang and J. S. Gutkind , “G Protein‐Coupled Receptors and Heterotrimeric G Proteins as Cancer Drivers,” FEBS Letters 594, no. 24 (2020): 4201–4232. 33270228 10.1002/1873-3468.14017 PMC8849590

[23] P. Langfelder and S. Horvath , “WGCNA: An R Package for Weighted Correlation Network Analysis,” BMC Bioinformatics 9 (2008): 559. 19114008 10.1186/1471-2105-9-559 PMC2631488

[24] Y. Chen , L. Liao , B. Wang , and Z. Wu , “Identification and Validation of Immune and Cuproptosis ‐ Related Genes for Diabetic Nephropathy by WGCNA and Machine Learning,” Frontiers in Immunology 15 (2024): 1332279. 38390317 10.3389/fimmu.2024.1332279 PMC10881670

[25] M. Xu , H. Zhou , P. Hu , et al., “Identification and Validation of Immune and Oxidative Stress‐Related Diagnostic Markers for Diabetic Nephropathy by WGCNA and Machine Learning,” Frontiers in Immunology 14 (2023): 1084531. 36911691 10.3389/fimmu.2023.1084531 PMC9992203

[26] C. Wei , Y. Wei , J. Cheng , et al., “Identification and Verification of Diagnostic Biomarkers in Recurrent Pregnancy Loss via Machine Learning Algorithm and WGCNA,” Frontiers in Immunology 14 (2023): 1241816. 37691920 10.3389/fimmu.2023.1241816 PMC10485775

[27] V. Giandomenico , T. Cui , L. Grimelius , K. Öberg , G. Pelosi , and A. V. Tsolakis , “Olfactory Receptor 51E1 as a Novel Target for Diagnosis in Somatostatin Receptor‐Negative Lung Carcinoids,” Journal of Molecular Endocrinology 51, no. 3 (2013): 277–286. 23969981 10.1530/JME-13-0144

[28] P. D. Stevens and B. O. Williams , “LGR4: Not Just for Wnt Anymore?,” Cancer Research 81, no. 17 (2021): 4397–4398. 34470783 10.1158/0008-5472.CAN-21-2266

[29] N. Krishnamurthy and R. Kurzrock , “Targeting the Wnt/Beta‐Catenin Pathway in Cancer: Update on Effectors and Inhibitors,” Cancer Treatment Reviews 62 (2018): 50–60. 29169144 10.1016/j.ctrv.2017.11.002 PMC5745276

[30] D. J. Stewart , “Wnt Signaling Pathway in Non‐Small Cell Lung Cancer,” Journal of the National Cancer Institute 106, no. 1 (2014): djt356. 24309006 10.1093/jnci/djt356

[31] Y. Dodin , “Identification of LGR4 as a Prognostic Biomarker in KRAS‐Mutant Lung Adenocarcinoma: Evidence From Integrated Bioinformatics Analysis,” Medicine 102, no. 46 (2023): e36084. 37986325 10.1097/MD.0000000000036084 PMC10659610

[32] X. Gong , J. Yi , K. S. Carmon , et al., “Aberrant RSPO3‐LGR4 Signaling in Keap1‐Deficient Lung Adenocarcinomas Promotes Tumor Aggressiveness,” Oncogene 34, no. 36 (2015): 4692–4701. 25531322 10.1038/onc.2014.417 PMC4476959

[33] H. Mohammadpour , C. R. MacDonald , P. L. McCarthy , S. I. Abrams , and E. A. Repasky , “β2‐Adrenergic Receptor Signaling Regulates Metabolic Pathways Critical to Myeloid‐Derived Suppressor Cell Function Within the TME,” Cell Reports 37, no. 4 (2021): 109883. 34706232 10.1016/j.celrep.2021.109883 PMC8601406

[34] G. Bruno , N. Nastasi , A. Subbiani , et al., “β3‐Adrenergic Receptor on Tumor‐Infiltrating Lymphocytes Sustains IFN‐γ‐Dependent PD‐L1 Expression and Impairs Anti‐Tumor Immunity in Neuroblastoma,” Cancer Gene Therapy 30, no. 6 (2023): 890–904. 36854895 10.1038/s41417-023-00599-x PMC10281870

[35] A. M. Globig , S. Zhao , J. Roginsky , et al., “The β(1)‐Adrenergic Receptor Links Sympathetic Nerves to T Cell Exhaustion,” Nature 622, no. 7982 (2023): 383–392. 37731001 10.1038/s41586-023-06568-6 PMC10871066

[36] S. Lehrer and P. H. Rheinstein , “The ADRB1 (Adrenoceptor Beta 1) and ADRB2 Genes Significantly Co‐Express With Commonly Mutated Genes in Prostate Cancer,” Discovery Medicine 30, no. 161 (2020): 163–171. 33593484 PMC7894950

[37] H. H. Lin , “Adhesion Family of G Protein‐Coupled Receptors and Cancer,” Chang Gung Medical Journal 35, no. 1 (2012): 15–27. 22483424 10.4103/2319-4170.106170

[38] Y. Wu , H. Liu , Z. Sun , et al., “The Adhesion‐GPCR ADGRF5 Fuels Breast Cancer Progression by Suppressing the MMP8‐Mediated Antitumorigenic Effects,” Cell Death & Disease 15, no. 6 (2024): 455. 38937435 10.1038/s41419-024-06855-8 PMC11211477

[39] M. Lv , X. Li , W. Tian , H. Yang , and B. Zhou , “ADGRD1 as a Potential Prognostic and Immunological Biomarker in Non‐Small‐Cell Lung Cancer,” BioMed Research International 2022 (2022): 5699892. 36457341 10.1155/2022/5699892 PMC9708333

[40] L. Yang , Z. Zhang , J. Dong , et al., “Multi‐Dimensional Characterization of Immunological Profiles in Small Cell Lung Cancer Uncovers Clinically Relevant Immune Subtypes With Distinct Prognoses and Therapeutic Vulnerabilities,” Pharmacological Research 194 (2023): 106844. 37392900 10.1016/j.phrs.2023.106844

[41] Z. Zhang , X. Sun , Y. Liu , et al., “Spatial Transcriptome‐Wide Profiling of Small Cell Lung Cancer Reveals Intra‐Tumoral Molecular and Subtype Heterogeneity,” Advanced Science (Weinheim, Baden‐Wurttemberg, Germany) 11, no. 31 (2024): e2402716. 38896789 10.1002/advs.202402716 PMC11336901

[42] A. Santoro , W. C. Su , A. Navarro , et al., “Phase Ib/II Study of Ceritinib in Combination With Ribociclib in Patients With ALK‐Rearranged Non‐Small Cell Lung Cancer,” Lung cancer (Amsterdam, Netherlands). 166 (2022): 170–177. 35298959 10.1016/j.lungcan.2022.02.010

[43] A. Qin , L. Wells , B. Malhotra , et al., “A Phase II Trial of Pevonedistat and Docetaxel in Patients With Previously Treated Advanced Non‐Small‐Cell Lung Cancer,” Clinical Lung Cancer 25, no. 2 (2024): 128–134. 37977950 10.1016/j.cllc.2023.10.011

[44] J. Kryczka , J. Kryczka , K. H. Czarnecka‐Chrebelska , and E. Brzeziańska‐Lasota , “Molecular Mechanisms of Chemoresistance Induced by Cisplatin in NSCLC Cancer Therapy,” International Journal of Molecular Sciences 22, no. 16 (2021): 8885. 34445588 10.3390/ijms22168885 PMC8396273

[45] M. Konoshenko , Y. Lansukhay , S. Krasilnikov , and P. Laktionov , “MicroRNAs as Predictors of Lung‐Cancer Resistance and Sensitivity to Cisplatin,” International Journal of Molecular Sciences 23, no. 14 (2022): 7594. 35886942 10.3390/ijms23147594 PMC9321818

