# Supplementary figures and images for: Therapeutic and Prognostic Potential of G Protein‐Coupled Receptors in Lung Adenocarcinoma: Evidence From Transcriptome Data and In Vitro Experiments
Source: Clin Respir J. 2025 May 13;19(5):e70080. doi: 10.1111/crj.70080 (PMC12075931; doi:10.1111/crj.70080)

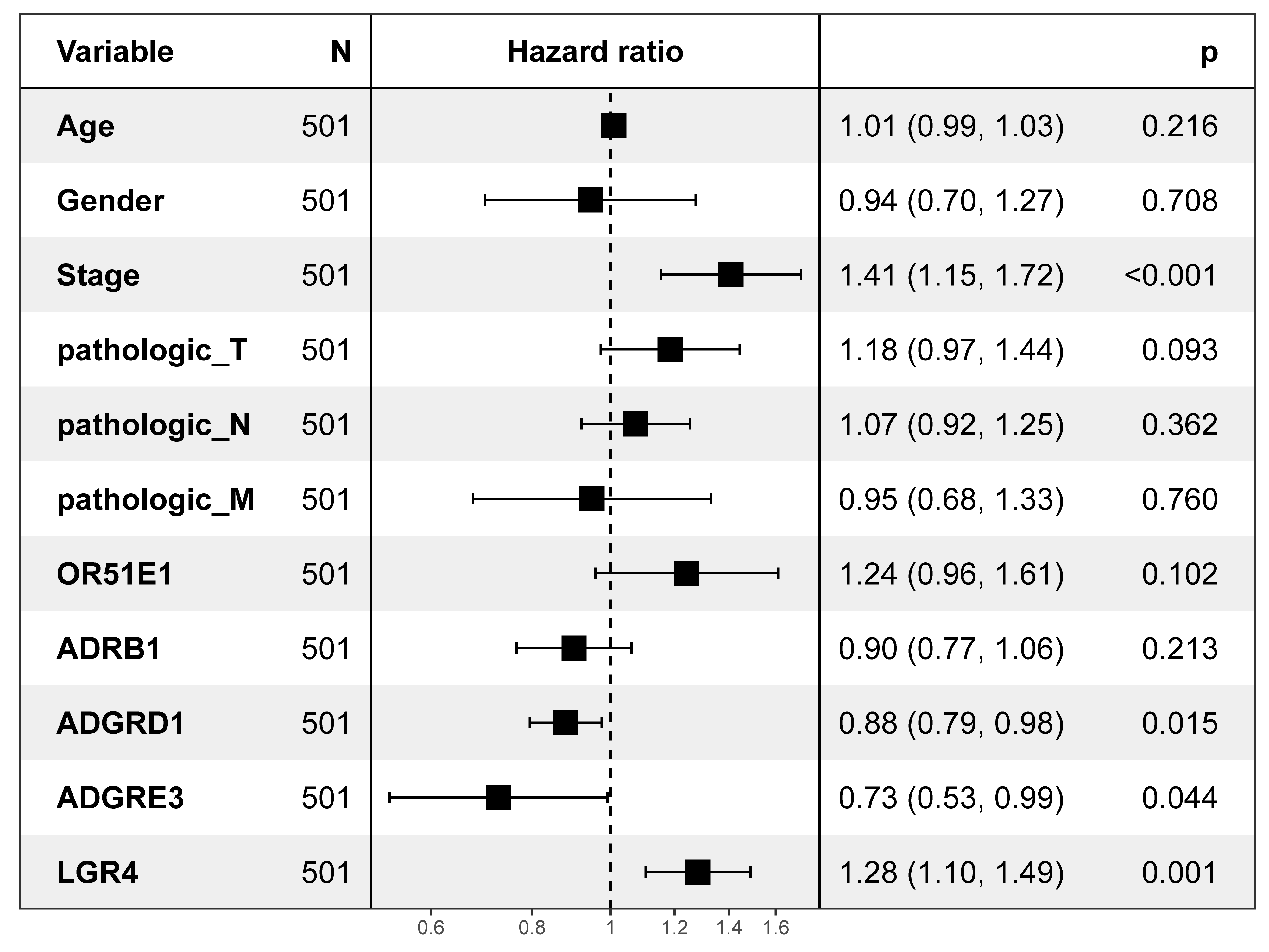

Supplement: Supplementary file 1 — Figure S1. Identification of independent prognostic factors by multivariate Cox regression. [file CRJ-19-e70080-s002.tif]
