# Supplementary material for: Therapeutic and Prognostic Potential of G Protein‐Coupled Receptors in Lung Adenocarcinoma: Evidence From Transcriptome Data and In Vitro Experiments
Source: Clin Respir J. 2025 May 13;19(5):e70080. doi: 10.1111/crj.70080 (PMC12075931; doi:10.1111/crj.70080)
Supplement: Supplementary file 2 — Table S1. Sequences of the primers used in qRT‐PCR. This integrated analysis demonstrates the involvements of G protein‐coupled receptors (GPCRs) in lung adenocarcinoma developments, and proves the potential of five key GPCRs as prognostic biomarkers and targets in lung adenocarcinoma. [file CRJ-19-e70080-s001.docx]

**Table S1. Sequences of the used primers in qRT-PCR**

| **Gene name** | **Sequences (5'-3')** | **Length (bp)** |
| --- | --- | --- |
| OR51E1 | TCCTACTGCCTACACCAAGA | 118 |
|  | AGGAGATGAGAAGTGAGTCCA |  |
| LGR4 | GCATCCCTGACTTTGCATTTAC | 117 |
|  | AGGTCTCCAGGTTATCTAGTCC |  |
| ADRB1 | ATCGCCTCGTCCGTAGT | 109 |
|  | CGCAGCTGTCGATCTTCTT |  |
| ADGRE3 | TGAGTCACTTCTCACCAATCAG | 93 |
|  | GATTCCACATCCCGGAGAATAG |  |
| ADGRD1 | CGTGCTCCTACACTACTTCTTC | 117 |
|  | TAGTAACGGTGCTTGCTGTC |  |
| GAPDH | CCTGCACCACCAACTGCTTA | 72 |
